# Forensic Nursing competencies in disasters situations: scoping review

**DOI:** 10.1590/1980-220X-REEUSP-2022-0486en

**Published:** 2023-09-25

**Authors:** Thiago Augusto Soares Monteiro da Silva, Débora Fernanda Haberland, Thais da Silva Kneodler, Alex Coelho da Silva Duarte, Joyce Williams, Alexandre Barbosa de Oliveira

**Affiliations:** 1 Universidade Federal do Rio de Janeiro, Escola de Enfermagem Anna Nery, Rio de Janeiro, RJ, Brazil.; 2 Stevenson University, Berman School of Nursing and Health Professions, Owings Mills, MD, USA.

**Keywords:** Forensic Nursing, Disasters, Professional Competence, Science of Disaster, Enfermagem Forense, Desastres, Competência Profissional, Ciência do Desastre, Enfermería Forense, Desastres, Competencia Profesional, Ciencia del Desastre

## Abstract

**Objective:**

To map sources of technical-scientific information on Forensic Nursing competencies in disasters situations.

**Method:**

Scoping review developed in accordance with the JBI methodology, carried out in three stages by two reviewers independently on selected databases and gray literature, using keywords and descriptors. After reading them in full, we proceeded with data extraction and content analysis of 28 publications.

**Results:**

Identified 24 publications in English, 20 of which were produced in the United States of America. 24 competencies of forensic nurses in disasters situations were mapped, including: training; resource management; link with the justice service; direct care; psycho-emotional care; collection and preservation of traces; registration and documentation with photography; body management; maintenance of the chain of custody; and epidemiological surveillance.

**Conclusion:**

The forensic nurse acts in all phases of disasters with the affected people, families and communities, under an interdisciplinary perspective. The development of competencies aimed at the practice of Forensic Nursing in disasters should be incorporated into the training strategies of these professionals, in order to obtain better response standards.

## RESUMO

**Objetivo:**

Mapear fontes de informações técnico-científicas relativas às competências da Enfermagem Forense em situações de desastres.

**Método:**

Revisão de escopo desenvolvida de acordo com a metodologia JBI, realizada em três etapas por dois revisores, de forma independente, em bases de dados e literatura cinzenta selecionadas, utilizando palavras-chave e descritores. Depois de lê-los na íntegra, foi feita extração de dados e análise de conteúdo de 28 publicações.

**Resultados:**

Identificamos 24 publicações em inglês, das quais 20 foram produzidas nos Estados Unidos da América. Foram mapeadas 24 competências do enfermeiro forense em situações de desastres, entre elas: treinamento; gerenciamento de recursos; vínculo com o serviço de justiça; atendimento direto; atendimento psicoemocional; coleta e preservação de vestígios; registro e documentação com fotografia; gerenciamento de corpos; manutenção da cadeia de custódia; e vigilância epidemiológica.

**Conclusão:**

O enfermeiro forense atua em todas as fases dos desastres com as pessoas, famílias e comunidades afetadas, em uma perspectiva interdisciplinar. O desenvolvimento de competências voltadas para a prática da Enfermagem Forense em desastres deve ser incorporado às estratégias de treinamento desses profissionais, a fim de se obter melhores padrões de resposta.

## RESUMEN

**Objetivo:**

Mapear las fuentes de información técnico-científica sobre competencias de Enfermería Forense en situaciones de catástrofes.

**Método:**

Scoping review desarrollada de acuerdo con la metodología del JBI, realizada en tres etapas por dos revisores de forma independiente sobre bases de datos y literatura gris seleccionadas, utilizando palabras clave y descriptores. Tras su lectura completa, se procedió a la extracción de datos y análisis de contenido de 28 publicaciones.

**Resultados:**

Se identificaron 24 publicaciones en inglés, 20 de ellas realizadas en Estados Unidos de América. Se mapearon 24 competencias de los enfermeros forenses en situaciones de catástrofes, entre ellas: formación; gestión de recursos; vínculo con el servicio de justicia; atención directa; atención psicoemocional; recogida y preservación de vestigios; registro y documentación con fotografía; gestión de cadáveres; mantenimiento de la cadena de custodia; y vigilancia epidemiológica.

**Conclusión:**

El personal de enfermería forense actúa en todas las fases de las catástrofes con las personas afectadas, familias y comunidades, en una perspectiva interdisciplinar. El desarrollo de competencias dirigidas a la práctica de la Enfermería Forense en catástrofes debe incorporarse a las estrategias de formación de estos profesionales, con el fin de obtener mejores estándares de respuesta.

## INTRODUCTION

Forensic Nursing is a science under advancement, as well as an art of caring that provides compassionate and culturally sensitive care to individuals, groups or communities who have experienced traumatic situations, violence, crimes, trafficking, disasters and ethical-legal situations that involve the care process of Nursing^(1,2,3,4)^.

This is an emerging specialty within global Nursing, whose scope is anchored in assistance, teaching, management and ­research on direct and indirect care for living or dead individuals who have experienced forensic situations, placed at the intersection between fundamental Nursing care, the legal-police services and the forensic sciences^(1,3,4,5)^. Such care is based on the principles of observation, evaluation/description, collection, preservation and storage of traces/evidence and forensic documentation/registration to achieve security and social justice^(1,3–5)^.

The specialty was consolidated in the 1980s, driven by the studies of Ann Wolbert Burgess and the conceptual model of integrated practice for the Science of Forensic Nursing, ­proposed by Virginia Lynch^(3,4,6,7)^. In 1991, Forensic Nursing was recognized by the American Academy of Forensic Sciences (AAFS) as one of the specialties belonging to the discipline of forensic sciences. The following year, in 1992, the International Association of Forensic Nurses (IAFN) was founded, through a meeting of 72 nurses in Minneapolis^(3,4,6,7)^.

Since then, the IAFN has been making efforts at the ­international level to disseminate scientific knowledge of Forensic Nursing and for updates about the specialty to ­several countries; this promoted the expansion of the scope and standards of practice of forensic nursing in accordance with the social, cultural, political, economic and philosophical context of Nursing and the legal system of each country where it is established^(1,4)^.

The dynamism of problematic situations that affect ­humanity, fostered the recognition of the need for the inclusion of forensic nurses in different contexts, such as in the care of individuals affected by sexual or interpersonal violence, in the prison environment, in psychiatric care, in the investigation of death, in human trafficking, in emergency units, in judicial expertise/technical assistance and, in disasters situations; such events being highlighted in the present study^(1,3–6,8)^.

In general, disasters refer to complex and multidimensional phenomena that emerge from natural and/or technological threats, the existence of human exposure and vulnerabilities, surpassing the capacity of a community to prepare and respond to the event using available resources^(9,10,11,12)^. It is commonly a public health problem and a threat to human rights, as it affects the environment and promotes the rupture of the pyramid of basic human needs of individuals, families and communities, causing physical, psycho-emotional and environmental damage in the short-, medium- and long-term^(9–12)^.

Thus, situations of a forensic nature emerge, such as violence against war victims, refugees, people in temporary shelters and field hospitals, physical and psycho-emotional trauma, suicide attempts, mass bodies without identification, abandonment and neglect of children, elderly and people with special needs, lack of structure in health and safety services. The need to ­comply with local, regional and/or national laws, different types of traces, as well as the reduced number of professionals and ­forensic scientists to act in the investigation processes of the event, may also produce legal consequences^(13)^.

In this complex context, the forensic nurse will be able to develop several actions and contributions in the pre-incident, incident and post-incident phases, which supports the urgent need to produce knowledge and define competencies, ­especially in disasters situations, to contribute to the advancement of scientific knowledge and the strengthening of the specialty. 

In view of the above, a preliminary search was carried out, in June 30, 2023, in MEDLINE (via PubMed), PROSPERO, Cochrane Database of Systematic Reviews and JBI Evidence Synthesis, in which no published or ongoing reviews on the competencies of Forensic Nursing in the context of disasters were evidenced, sustaining the need for this scoping review study, with the premise of mapping concepts in a systematized way.

Then, this study aimed to map sources of technical-scientific information on Forensic Nursing competencies in disasters situations.

## METHOD

This is a scoping review that followed the guidelines of the JBI^(14)^ methodology and the Preferred Reporting Items for Systematic Reviews and Meta-Analyses Extension for Scoping Reviews (PRISMA-ScR)^(15)^ checklist. In this sense, the ­research protocol was registered in the Open Science Framework (https://osf.io/3zxv7) with DOI 10.17605/OSF.IO/3WYSP. The protocol for this scoping review has been published and can be accessed via the following DOI 10.17665/1676-4285.20236615.

### Review Question

“What are the competencies of the forensic nurse in ­disasters situations?”

### Eligibility Criteria

The studies incorporated in this review were identified and selected based on the PCC mnemonic: Population, Concept, and Context. It is worth noting that the review question, ­objective, descriptors and keywords were aligned with the PCC mnemonic. Then, they were defined as:

Population – comprised nurses with knowledge or experience in disaster situations. Studies were included that deal with Forensic Nursing. This study understood that Forensic Nursing is a scientific discipline and an art that focuses on caring for individuals who are victims of violence, traumatic situations, ­crimes, trafficking, disasters and ethical-legal situations that involve the Nursing care process. This science under construction has the pillar that these situations lead to the intersection between health and justice services with the premise of achieving social justice and compassionate care^(1,3,4)^.

Concept – The included studies dealt with professional ­competence, which is the ability to plan, mobilize, integrate and transfer knowledge, skills, attitudes and resources to know how to act and interact in a complex and dynamic situation^(16)^.

Context – this review considered the context of disasters, whether of any type: technological, natural or social^(9–13)^. This review understood disasters situations as any serious public health problem as well as threats to human rights. It comprises a phenomenon that emerges from natural and/or technological threats, human exposure, vulnerabilities exceeding the capacity of a community to prepare and respond to the phenomenon using its resources. Studies that dealt with any type of disaster were considered for inclusion, namely: earthquakes, volcanic eruptions, landslides, tsunamis, avalanches, floods, heat and cold waves, forest fires, droughts, cyclones, epidemics and pandemics, technological risks and biological, hail, but not ­limited to these^(9–13)^.

It is worth bearing in mind that temporal and idiomatic clippings or open access definition were not marked out, in order to amplify the possibilities of recovering sources. The implementation of this review also had the participation of a third reviewer, to resolve conflicts, and with the support of a librarian.

With regard to sources of technical-scientific ­information, different types of publications were considered, including ­primary studies, with a quantitative, qualitative and mixed approach, experimental and quasi-experimental designs, case-control, reviews, before-and-after, time series, ­observational, cohort and cross-sectional studies. Gray literature was also considered (banks of theses and dissertations, websites, protocols, procedures, guidelines, books, book chapter, legislation, letters, opinions).

### Search Strategy

The searches were carried out between January 2022 to June 2023, in three stages, by two independent researchers, maintaining the blinding process. The searches for sources took place in three stages: the first was a primary search in the Medical Literature Analysis and Retrieval System Online (Medline) by way of Pubmed (carried out on July 5, 2023) and Cumulative Index to Nursing and Allied Health Literature (CINAHL) by way of EBSCOHost (carried out on July 5, 2023). In the second stage, a complete search was carried out in the databases, applying and adapting the keywords and descriptors together with the relationship of the Boolean operators “AND” and “OR”, It´s noteworthy that the identification considered separating the terms in Portuguese, Spanish and French for the VHL databases and in English for the international databases.

**Chart 1:**
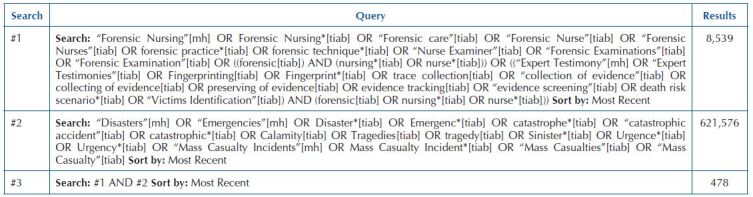
Search strategy in the Medical Literature Analysis and Retrieval System Online (Medline) by way of Pubmed – Rio de Janeiro, RJ, Brazil, 2023.

**Source:** Prepared by the authors, 2023.

The search strategy used in this review used the ­following descriptors and keywords: (“Forensic Nursing”[mh] OR Forensic Nursing*[tiab] OR “Forensic care”[tiab] OR “Forensic Nurse”[tiab] OR “Forensic Nurses”[tiab] OR forensic practice*[tiab] OR forensic technique*[tiab] OR “Nurse Examiner”[tiab] OR “Forensic Examinations”[tiab] OR “Forensic Examination”[tiab] OR ((forensic[tiab]) AND (nursing*[tiab] OR nurse*[tiab])) OR ((“Expert Testimony”[mh] OR “Expert Testimonies”[tiab] OR Fingerprinting[tiab] OR Fingerprint*[tiab] OR trace collection[tiab] OR “collection of evidence”[tiab] OR collecting of evidence[tiab] OR preserving of evidence[tiab] OR evidence tracking[tiab] OR “evidence screening”[tiab] OR death risk scenario*[tiab] OR “Victims Identification”[tiab]) AND (forensic[tiab] OR nursing*[tiab] OR nurse*[tiab]))) AND (“Disasters”[mh] OR “Emergencies”[mh] OR Disaster*[tiab] OR Emergenc*[tiab] OR catastrophe*[tiab] OR “catastrophic accident”[tiab] OR catastrophic*[tiab] OR Calamity[tiab] OR Tragedies[tiab] OR tragedy[tiab] OR Sinister*[tiab] OR Urgence*[tiab] OR Urgency*[tiab] OR “Mass Casualty Incidents”[mh] OR Mass Casualty Incident*[tiab] OR “Mass Casualties”[tiab] OR “Mass Casualty”[tiab]). Chart 1 presents the search strategy in the Medical Literature Analysis and Retrieval System Online (Medline) by way of Pubmed (carried out on July 5, 2023).

In the third stage, the reference lists of selected publications were analyzed to identify possible sources not retrieved by the search strategy, in accordance with the eligibility criteria, in addition to contacting the authors of the studies to obtain more information.

### Sources of Evidence

The searches were carried out in the following databases: Latin American and Caribbean Health Sciences Literature (Lilacs); Índice Bibliográfico Espanhol de Ciências da Saúde (IBECS); Bases de Dados de Enfermagem (BDENF); Red Peruana de Bibliotecas em Salud (LIPECS); Medline via PubMed of the National Library of Medicine (NLM); and the Scientific Electronic Library Online (Scielo). Through the VHL Regional Portal Information and Knowledge for Health, under the responsibility of the Latin American and Caribbean Center on Health Sciences Information (BIREME), searches were carried out in the Elsevier databases: Embase and Scopus, Clarivate Analytics: Web of Science, Ebsco: Cummulative Index to Nursing and Allied Health Literature (CINAHL) and Academic Search Premier (ASP). Also, through the Capes Journal Portal, the Comunidade Acadêmica Federada (CAFe) and CAB Direct, a platform that allows research in CAB Abstracts and Global Health. Also searched was Epistemonikos: Database of Best Evidence-Based Health Care, Information Technologies as well as through a network of experts.

The searches also included sites on Forensic Nursing, American Academy of Forensic Sciences (AAFS), Academy of Forensic Nursing (AFN), Canadian Forensic Nurses Association (CFNA), The UK Association of Forensic Nurses & Paramedics, Indo Pacific Academy of Forensic Nursing Science, Japan Association of Forensic Nursing, Sociedade Brasileira de Enfermagem Forense (SOBEF), Associação Brasileira de Enfermagem Forense (ABEFORENSE), Associação Portuguesa de Enfermagem Forense (APEFORENSE), Forensic Nursing Network Inc., professional legislation, the International Council of Nurses, the International Association of Forensic Nurses (IAFN), digital libraries of theses and dissertations, and internet search engines.

With regard to gray literature searches, access was through the gray literature and integration portal Science.gov: USA.gov, National Institute for Health and Care Excellence (NICE).

### Source Selection

After the searches, to organize the references and identify duplicate studies, the EndNote Web reference manager (Clarivate Analytics, PA, USA) was used. The texts were archived in digital folders. Please note, all identified citations were imported into the Rayyan application (Qatar Computing Research Institute, Doha, Qatar). The sources were analyzed by two reviewers, regarding titles, abstracts and descriptors, in accordance with the eligibility criteria. The excluded studies were registered and the reasons described. The source selection stage was carried out by two independent researchers, maintaining the blinding process. The execution of this stage also had the participation of a third reviewer, for conflict resolution, and with the support of a librarian.

### Data Extraction

After reading the full publications, data were extracted using an Excel tool, which was adapted in accordance with the JBI^(14)^ methodology, as follows: publication characterization data (authors, title, year, language, descriptors, source of information); origin and typology of the disaster situation; and competencies of Forensic Nursing in disaster prevention/mitigation, preparedness, response, recovery/rehabilitation. The data extraction step was performed by two independent researchers, maintaining the blinding process. The execution of this stage also had the participation of a third reviewer, for conflict resolution, and with the support of a librarian.

### Data Analysis and Presentation

After extracting the data, we proceeded with the inductive content analysis with the support of the software Interface de R pour les Analyses Multidimensionnelles de Textes et de Questionnaires (IRaMuTeQ). The results was presented in the form of a diagram, flow and chart, seeking the proper alignments with the objective and the research question. It should be noted that a descriptive presentation accompanies the mapped results.

## RESULTS

**Figure 1:**
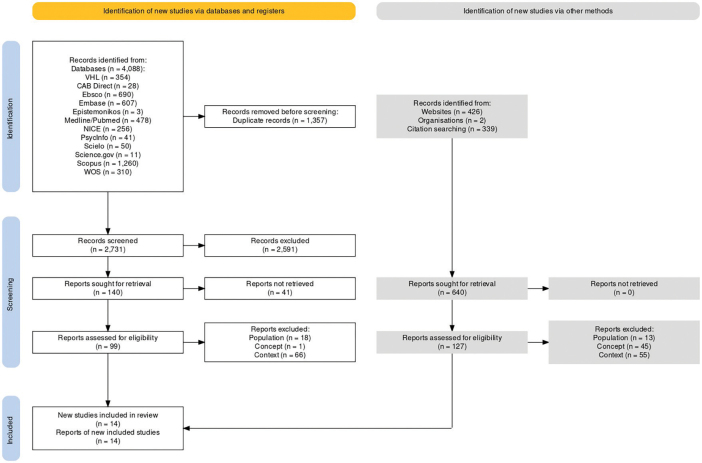
Flowchart of the selection stages of publications for the scoping review, adapted from the PRISMA-ScR model, 2023.

Identification of new studies via databases and registers a total of 4,088 publications were identified (BVS: 354; CAB Direct: 28; Ebsco: 690; Embase: 607; Epistemonikos: 03; Medline/Pubmed: 478; NICE: 256; PsycInfo: 41; Scielo: 50; Science.gov: 11; Scopus: 1,260; WOS: 310). Of these, 1,357 were excluded because they were duplicated (Figure 1).

The publications were exported to the Rayyan application, pairing and blinding the review of titles, abstracts and descriptors of 2,731 publications. Of these, 2,591 were excluded for not meeting the eligibility criteria, leaving 140 for full-text search and analysis. It is noteworthy that 41 publications were not retrieved, leaving 99 studies for the eligibility analysis. Of these, 66 were excluded for not meeting the context, 18 because the population, and one due to the concept. Thus, 14 publications were included via databases and registers (Figure 1).

Identification of new studies via other methods a total of 767, records identified from: websites: 426; organizations: 2; citation searching: 339. Reports sought for retrieval: 640, reports assessed for eligibility: 127. Of these, 55 were excluded for not meeting the context, 13 because the population, and 45 due to the concept. Thus, 14 publications were reports of newly included studies via other methods. After the identification and screening steps, a total of 28 publications were included for the final analysis (Figure 1).

As for the year, it was identified that the first publication was carried out in 1995^(17)^, two new publications in 2001^(20)^, one more in 2003^(21)^ and another in 2004^(22)^, three publications in 2005^(38)^ one more was found, four in 2020^(43)^ and one in 2022^(44)^.

With regard to language, 24 were published in English^(38)^. As for the location, 20 publications were ­produced in the United States of America (USA)^(17–33,35,40,44)^, three in Brazil^(36,42,43)^, three in India^(34,39–41)^ and two in Japan^(37,38)^.

**Chart 2:**
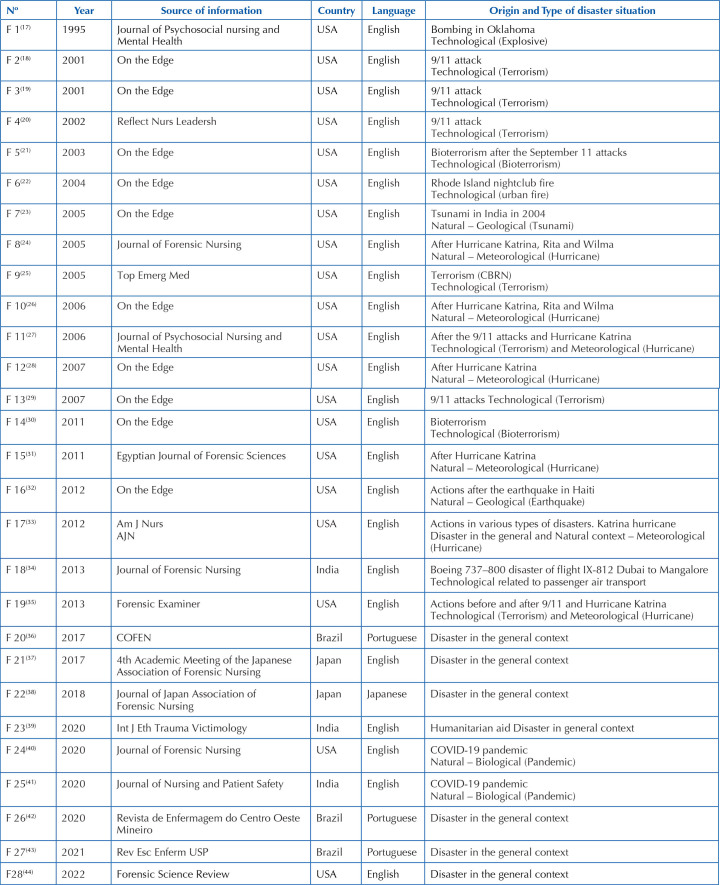
Characterization of publications included in the review (n = 28) – Rio de Janeiro, RJ, Brazil, 2023.

**Source:** Prepared by the authors, 2023.

The journals with the most publications on the subject were the newsletter On The Edge, with 10 publications^(18,19,21–23,26,28–30,32)^; the Journal of Forensic Nursing, with three publications^(24,34,40)^ and two publications in the Journal of Psychosocial Nursing and Mental Health^(17,27)^ (Chart 2).

Regarding the origin of the disaster, it was identified that a publication occurred after the Oklahoma attack in 1995^(17)^, four after the September 11, 2001 attacks^(22)^; tsunami in India, in 2004^(23)^; earthquake in Haiti, in 2010^(32)^; disaster with the Boeing 737–800, flight IX-812, Dubai to Mangalore, in 2010^(34)^. It was identified that two publications occurred after bioterrorism with Anthrax^(25)^ disasters, one in the context of humanitarian missions^(39)^, six in a general context of disasters^(36–38,42–44)^ and two after the September 11 attack and the occurrence of Hurricane Katrina^(27,35)^.

With regard to the typology of disasters, 10 publications^(22)^, one about explosives^(17)^ and one about air transportation^(34)^. In the natural typology, nine publications were identified^(23,24,26,28,31–33,40,41)^, divided into five on meteorological disasters^(39)^.

**Figure 2:**
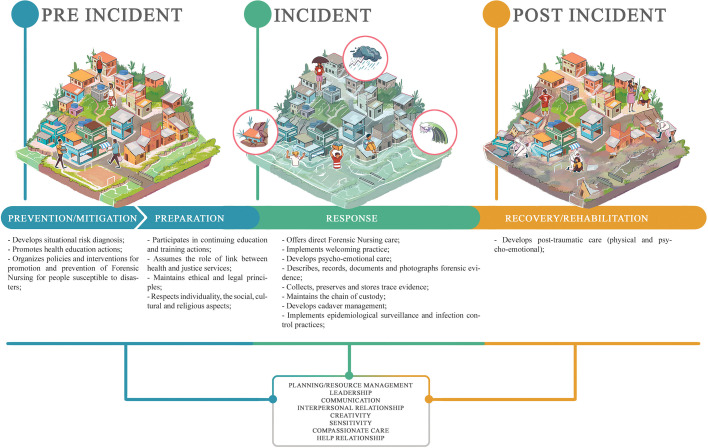
Mapping of Forensic Nursing competencies in the context of Disasters, 2023.

As for the mapping of forensic nurses competencies in disasters situations, 24 competencies were identified ­listing the three phases of disasters (pre-incident, incident and post-incident), as shown in Diagram (Figure 2). Of these, leadership^(18,20,24,25,31–33,35,44)^, communication^(18,20–22,24–28,31,33,35,38,39,43)^, creativity^(18,23–25,28,35,41)^, sensitivity^(28,41,44)^, interpersonal relationship (teamwork and relationship with patients and family members)^(21–28,30,31,33,35–37,39,40,42–44)^, the compassionate care^(22,24,37,39,44)^ and the helping relationship (holistic)^(18–20,22,24,27,33,36,39–41,44)^, as well as the planning/management of resources (predictive assessment)^(18–28,31–37,39,40,42,44)^ were identified in publications as essential in the three phases of disasters and comprise personal and relational aspects of the forensic nurse.

With regard to the competencies of the forensic nurse in the pre-incident phase (prevention/mitigation and preparation), the most noteworthy competencies comprise the situational diagnosis of risks (environmental and human)^(18,23,25,27,28,32,33,35–40,44)^, health education^(21,31,32,35,37,38,44)^ and the promotion/prevention policies and interventions for people susceptible to disasters^(21,37,42)^. In the preparation phase, competencies were identified involving permanent education and training^(17–28,30–33,35,37,39,42,44)^, the link with the justice service^(17,21,25,27,35–37,40,41,43)^, respect for individuality and socio-cultural and religious aspects^(24,25,32,33,35,37,39,44)^ and, maintenance of ethical and legal principles^(21,25,27,28,32,39,43,44)^.

In the incident response phase (response), direct care stood out with the competencies of Forensic Nursing (interview, forensic physical examination, basic and advanced trauma life support care)^(17–25,27,28,30–41,44)^, welcoming^(17,19,22,24,25,27,35,39,41,43)^, psycho-emotional care (emotional support for patients, family members, teams through prayer services, games with children, rest for professionals)^(18–24,27,29,30,32,33,35–37,39)^, the description, registration and documentation of persons with photography^(17,21,22,25,30–32,34,35,37,39,40,43,44)^, collection, preservation and storage of traces^(17,21,22,24,25,28,30,32,33,35–40,43,44)^, maintenance of the chain of custody^(17,21,25,43)^, identification and management of dead bodies^(22,31,34–39,44)^, infection control, and epidemiological surveillance^(21,25,26,31,34,37–40,43)^.

In the post-incident phase (recovery/rehabilitation), the post-traumatic care competency (physical, behavioral surveillance and neurobiology of trauma) was highlighted^(18–21,24,27,29,31,35)^.

It is worth noting that, due to the dynamism of disaster situations, the competencies of Forensic Nursing are presented in a continuum. Therefore, some are repeated in more than one phase and denote that the professional may be working in one phase while another begins or is occurring concomitantly. As an example of these competencies that overlap throughout the phases, we have: resource planning/management; leadership; communication; interpersonal relationship; creativity; sensitivity; compassionate care; and helping relationship.

## DISCUSSION

Disaster situations can lead individuals or families to the courts of justice to seek the fulfillment of their rights through compensation for material, physical and psycho-emotional losses, or they can determine law operators to search for traces and records of care provided to victims, with the premise of contributing to the investigative process^(17,43,44)^. Thus, the presence of forensic nurses in the context of disasters can make substantial contributions to support the investigation process and legal conduct for victims, as Nursing is the largest category of health professionals and nurses are often the first responders in disasters situations^(31,39,44)^.

Indeed, studies^(45–47)^ point out that victims of epidemics, air disasters and those with post-traumatic stress resulting from wars can enter emergency units and, as they are potential forensic cases, will need the care provided by the nurses, who need to use a forensic lens to identify traces. Such professionals must possess a theoretical-practical framework to collect, preserve and store traces and contribute to the scientific investigation of catastrophic deaths, promoting an intersection of Nursing care with the legal system, in order to fulfill the mission of reaching the forensic needs of individuals and social justice^(45,46,47)^.

Despite the notes of some theorists on the need for inclusion of forensic nurses in disaster situations, the review could not find studies that dug deeper on which competencies would be appropriate in this context. The importance of this forensic professional began to be considered only from 1995^(17)^, after the attack in Oklahoma, when certain notes were addressed in a publication on how the forensic nurse could contribute in the context of disasters, highlighting the need for a response in a quick and qualified way through the competencies of reception, description of injuries, identification, collection and preservation of forensic traces, and interaction with the operators of the legal system^(17)^.

Through this scoping review, it was evident that the interest in this topic is incipient and has been reactively presented in scientific productions, since most publications were carried out shortly after the occurrence of major disaster situations^(35,48)^. It is mentioned, for example, that on the subject of terrorism there were publications^(18–21,25)^ in 2001, 2002, 2003 and 2005, close to the attacks of September 11, 2001; however, there was a decrease in productions on the subject over the following years.

It should be noted that in 2001, after the September 11 attacks, the IAFN published resolution I in the newsletter On the Edge, which condemned any terrorist event against humanity and requested the inclusion of disaster preparedness in the scope of training of forensic nurses, leaving the proposal to offer an education program to be discussed at the 2002 IAFN Scientific Assembly^(19,49)^.

With regard to the source of information, it was identified that the main mediums of dissemination were On the Edge (OTE) and the Journal of Forensic Nursing (JFN), both from the International Association of Forensic Nurses (IAFN). The OTE was a newsletter that disseminated information about Forensic Nursing from 1995 to 2012. The newspaper stood out as an important means of disseminating the practice of the specialty internationally through articles, experience reports, interviews, events and other information^(50,51,52)^.

The JFN represents a vehicle for disseminating scientific knowledge obtained through research, which follows methodological rigor to consolidate the science of Forensic Nursing, a science that is experimenting continued growth^(50–52)^.

It should emphasized that, in a study on the state of the science of Forensic Nursing, published in 2020, an analysis of publications in the JFN was carried out in the time frame from 2005 to 2019, where there was a predominance of publications on sexual assault, violence against children, the elderly and intimate partners, noting a reduction of productions in the ­category that addressed themes related to disasters, risk ­management and policies^(52)^. Thus, there is an urgent need for more contributions and discussions that can investigate the gaps in scientific knowledge of the specialty in the context of disasters.

Despite the advances in Forensic Nursing, with the dissemination to several countries in the world, through this review it was identified that the USA and the English language had the highest frequency. This result may be related to the fact that the birth of the specialty occurred in the USA, as it is the host country of the IAFN and also due to the fact that several disasters have occurred in that country, which served as a background for the productions on the insertion of Forensic Nursing in such contexts, pointing out the authors concern to contribute with reflections and experiences with a view to consolidating the scope and standards of practice for the response of the forensic nurse.

It was observed that the most recent publications were from countries such as India, Japan and Brazil, where Forensic Nursing has a shorter implementation time, being countries that have a history of complex disasters that affected the population with different types of damage.

It is noteworthy that each country has its specificities with regard to the performance of Forensic Nursing, the legal system and the typology of disasters, supporting the need for investments in teaching, research and extension in order to clarify how the forensic nurse can be inserted in this context, how they can contribute to individuals affected by disasters and to legal operators.

The study showed that most publications referred to disasters of a technological nature, highlighting terrorism/bioterrorism^(21,25,30)^, and meteorological natural disasters caused by hurricanes; however, few addressed CBRN events, which points to the existence of knowledge gaps, supporting the need for more studies that can contribute with robust scientific information on the competencies of forensic nurses in disasters.

The 24 Forensic Nursing competencies listed in the publications support nursing practice standards in the three stages of disasters: pre-incident, incident and post-incident. In the pre-incident phase, the forensic nurse can contribute to care for the prevention/mitigation of risks to individuals and communities by performing the situational risk diagnosis, resulting in prevention for the population and professionals^(35)^.

Therefore, forensic nurses can, for example, work in the field of Public Health through the competence of health ­education^(40)^.

In order to ensure that the insertion and performance of forensic nurses may occur in a safe and effective way, it is necessary the competence of permanent education with training on how to identify, anticipate situations and act in adverse scenarios and with few resources, what care to perform and how to solve conflicts in a context of instability, insecurity and unpredictability^(17,19–22,24–36–40,42,44)^.

There is a need to include the theoretical-practical theme with realistic simulations in the context of training forensic nurses with performance evaluation^(28)^. To this end, it is pointed out, through the competencies listed in this study, that training should include the concepts and typologies of disasters, such as the identification of risk situations and CBRN events^(21)^, ethical-legal aspects, risk management actions, management of Forensic Nursing care in disasters, reception, interview and forensic physical examination, protocols for collection, preservation and storage of traces in disasters, isolation care, decontamination, treatment and vaccination, personal protective equipment, forensic records and photo documentation, chain of custody in disasters, management of dead bodies, theory and principles of Forensic Nursing, neurobiology of trauma and psycho-emotional care^(17–44)^. It is argued that certified courses would enable forensic nurses to have autonomy and, professional and legal recognition to work in this context.

It is noted that permanent education with training includes, in the pre-incident phase, the identification of risks and primary prevention to prepare nurses to act in the face of an event, and in the post-incident phase, to learn from the lessons arising from the disasters and promote evolutionary leaps for Forensic Nursing care effectively in future events^(18,26–28,35,37,43)^. It is mentioned, for example, that as a result of Hurricane Katrina, the IAFN set up an ad hoc committee to analyze disaster planning, promoting the discussion of the need for new procedures and guidelines^(18,26–28,35,37,43)^.

In addition forensic nurses can contribute to the development of policies and interventions for promotion/prevention for people susceptible to disasters by proposing, together with public health managers, engineers, biologists, legal operators and other members of the multidisciplinary team, guidelines, protocols, multi-risk and contingency plans with continuous updates^(21,25,28–30,32,35–37,39,40,43,44)^.

It is argued that, in order to allow forensic nurses to be inserted in this context, it is necessary to create a link between the Nursing and the justice services, in order to provide a legal framework to respond to disasters with safety and autonomy and guided by the deontological code of the profession. They must, therefore, be in compliance with the professional council, and respect local, regional and federal laws^(43)^. In addition, forensic nurses must know the geography of the disaster site and adapt to the social, cultural and religious aspects of the population to support their forensic care with respect for the individuality and integrity of people affected by disasters^(17,21,24–27,32,35–37,39–41)^.

This competence reduces the possibilities of secondary victimization, strengthens the interpersonal relationship of the forensic nurse with the community so that the reception and direct care can be carried out and contributes effectively so that the families have assistance to the right care and cultural and religious rites in the preparation and burial of the bodies of individuals affected by disasters^(22)^.

In the response phase, forensic nurses contribute to risk management for survivors who are at the disaster site, aiming to identify the risk of violence, negligence, abandonment of children and the elderly, as well as to perceiving the risks to which health professionals may be exposed to^(18,20,23,25,30,33,35,36,38–41,44)^. There are risks of landslides, falling poles, exposed wires, chemical, biological, radiological or nuclear contamination^(20,23,25,30,33,35,36,38–41,44)^. Safety is paramount for all scenarios. Thus, the professional will use observation of the environment and communication with the incident chain of command to identify, notify, and plan the team’s performance in a safe way.

People affected by disasters will need reception and direct care from the forensic nurse through screening, interview and forensic physical examination, decontamination, evacuation, admission, notification, application of the Forensic Nursing process, in addition to prioritize basic and advanced life support^(17,19–25,27,28,30–33,35–41,44)^. This care is guided by the ability to plan/manage resources in a flexible way to meet identified needs and must be articulated with the activities performed by the multidisciplinary team^(17,19–25,27,28,30–33,35–41,44)^.

It is mentioned that, when the disaster victim is received, the interview should be carried out as soon as possible^(25)^ and the psycho-emotional impacts assessed, such as anxiety, anguish, fear, hallucinations, delusions, feelings of insecurity, suicidal ideation, anger and emotional shock^(18–24,29,30,32,33,35,36,39,41)^. In turn, the members of the teams involved may experience tiredness, changes in the circadian cycle, vicarious trauma and emotional exhaustion^(18–24,29,30,32,33,35,36,39,41)^. In this sense, the forensic nurse must offer psycho-emotional support care to patients, families and teams, through psycho-spiritual comfort, by organizing prayer services, contacting family members to reassure them, promoting games and interactive activities with children, making possible for professionals to rest and, provide active listening^(18–24,29,30,32,33,35,36,39,41)^. Forensic nurses who respond must also assess their anxiety, emotional and physical stresses and take appropriate actions, taking breaks from the scene knowing that human factors can impact the overall outcome for survivors and personnel.

Furthermore, one of the ways of caring performed by forensic nurses^(32)^ to meet the forensic needs of individuals is through the competence of collecting, preserving and storing traces. Nurses should be aware of the fact that disasters possibly represent a large and complex crime scene and that victims may have traces on their bodies that will help law enforcement officers to understand the event and its repercussions and, thus, enable victims to achieve social justice and obtain compensation for damages caused by the event. In this way, during the direct care of Forensic Nursing, attention should be paid to the identification of wires, metals, screws, clothes, fibers, earth, measurement of chemical, biological, radiological and nuclear waste, which can contribute to legal investigation after the occurrence of events^(17,21,22,25,28,30–33,35–40,43,44)^.

Collecting, preserving and storing forensic traces can serve as a link to identify the modus operandi, the devices used and the perpetrator^(25)^ and look for the presence of traces in the body of individuals victims of disasters.

It is important to note that, this collection, preservation and storage takes place in a complex scenario due to the existence of multiple traces, which can be real or illusory. In addition, the need for decontamination in CBRN events can lead to the loss of traces, making it necessary to measure the radiation level of the victims before decontamination, with the collection of nasopharyngeal swabs (keeping them separate from each other), fluids (sputum, vomit, blood, urine, feces, thoracic drainage and bronchial lavage) and photo documentation^(21,25)^.

Among some actions, the need to keep the victim ­standing on a white sheet to remove the clothes, which must be packed and double-bagged in sealed and labeled impermeable containers^(25)^; perform radiographs, keep the patient away from sources of electrical current and defibrillators^(25)^; collect micro traces, and keep them packed and labeled^(25)^. It is suggested the use of cell phone applications that present QR-code or barcode, which allow the recording of professional data, destination, date, time, names on the swabs. Most studies point out that the forensic nurse will be able to collect, preserve and store traces, even though they do not point out which traces, how to collect them safely, where to store them and for how long.

Another problem involves the breakdown of health services and the loss of supplies, which reinforces the need for nurses to perform care with creativity, flexibility and dynamism. Thus, the development of light collection kits, easy to handle and move, can contribute in this context^(25,30)^.

Thus, it is necessary to plan where these traces will be stored, how they will be delivered to the crime labs and how to act in the event of an interruption in the supply of electricity and communication, and how the forensic nurse will be able to act to maintain the chain of custody in this complex scenario and sustain the legal suitability of the traces in order to maintain the safety of the environment, professionals and third parties^(25,43)^. It is pointed out that it would be appropriate to have the presence of a professional to monitor the traces, the elaboration of protocols, training and legislation to support this competence of the forensic nurse^(21,25,53)^.

It should be noted that the collection of traces and the investigation of the dynamics of the facts at the site of the accident is an act performed by the criminal experts who, depending on the country, may have nurses on the team. It is up to the forensic nurse to collect forensic traces in care spaces, such as field hospitals, emergency units, hospital clinics, intensive care units, among others.

All care must be described, being attentive to register and document the number of injuries and the affected sites in ­physical or digital records, using body diagrams, in ­addition to the main complaint and health history and, if possible, ­complemented with photographs of physical injuries and ­traces collected, maintaining a line of surveillance based on the privacy and confidentiality of the information collected and the link with legal and criminalistics services when transmitting the collected traces^(21,25,28,30–32,37,39,43,44)^.

Currently, the use of cameras and applications on cell phones facilitates the storage and transmission, as well as the registration and resolution of doubts about information during a disaster situation in a practical and easy-to-handle way, strengthening communication with other professionals and with affected individuals^(21)^. The use of technologies that work offline can contribute to overcoming obstacles in forensic recording^(32)^.

The forensic nurse also contributes to the management of deceased and/or body parts after the event, by preparing mobile mortuary facilities/morgues to receive the corpses and/or remains and personal objects, collecting samples of deoxyribonucleic acid (DNA), assisting the mortuary team performing body preparation care for medical-legal evaluation and for the returning to the family environment^(21,22,24,31,33,34,36–39,44,54)^. This competence helps the family to continue the symbolic rites, in accordance with their culture and religion^(21,22,24,31,33,34,36–39,44)^.

The forensic nurse contributes to the ante-mortem and/or post-mortem interview, obtaining, personally or by telephone with the family members, information on the description of the individuals’ distinctive characters, health history, radiographic examinations, dental treatment, personal objects, work history, when scanning photographs and identifying signs of death^(22,39)^. Another fact is that the management of cadavers can contribute to their identification, to avoid exchange due to identification or release errors, to evaluate the environmental conditions that can lead to changes in the body (temperature, water, oxygen levels, depth)^(38,39)^ and to control infections and the spread of disease. It is emphasized that this care must be carried out in an integrated manner with the team of criminal experts and that the inclusion of the forensic nurse is beneficial because it presents, in its essence, the competence of sensitivity, in addition to having knowledge about anatomy, physiology, pathology, pharmacology, criminalistics, ethics, legislation and forensic practices^(21,22,31,33,34,36–40,43,44,54)^.

As for the control and dissemination of infections and epidemiological surveillance, the forensic nurse contributes through telemonitoring, guiding on signs and symptoms, forms of transmission and on care for prevention and treatment, in addition to forensic issues that emerge from outbreaks, epidemics and pandemics^(26,31,33,36)^.

In the recovery/rehabilitation phase, the forensic nurse ­performs competencies by carrying out post-traumatic care for the physical and psycho-emotional scope of individuals who have experienced a disaster situation and who are recovering from burns, amputations, injuries, post-traumatic stress disorder (PTSD), anxiety, depression, among other physical and psycho-emotional health deviations commonly seen in these contexts^(29,31,32,35,54)^.

With regard to the essential competencies in both phases of the disaster management continuum, it was evident that resource planning/management was listed by most publications, since, in the face of disaster situations, a complexity of problems arising from the breakdown of health, safety and communication services, loss or shortage of materials, equipment, low financial, nutritional, sanitation and water supply resources especially when working in austere conditions^(18,20–24,32)^.

Thus, the nurse contributes by leading, establishing interpersonal relationships with individuals, families and teams of professionals from pre-hospital and emergency care units, hospital clinics and the police, to plan care and organize care spaces, select the appropriate location for setting up field hospitals and family assistance centers, searching for appropriate means of communication linked to a chain of command, logistically forecasting the use of supplies and personal protective equipment in an economical way, provision of antibiotics, dressings, medicines and blood supplies, performing backup records and documents, organizing work schedules, reorganizing outpatient demands and elective surgeries. This professional also deals with climatic adversities, manages the flow of people and objects, maintaining creativity and sensitivity (through the ability to feel, think and act) to promote compassionate care and establish a helping relationship with the affected people, providing continuity of care, interrelating care scenarios and/or disasters scenarios with the courts of justice^(18,20–28,30–33,35–44,54)^.

As a consequence of the above, that the role of the forensic nurse in the context of disasters is complex and transcends the collection of traces to preserve crime scenes. It is recommended that more studies be carried out on Forensic Nursing care models in this context^(24,28,39,42,44)^.

It is understood that this review has the potential to ­contribute to the conduction of more research on the science of Forensic Nursing, expanding the dissemination of the scope and standards of practice of the specialty within undergraduate and graduate courses, which may help in the definition and adoption of guidelines for the training of forensic nurses.

The study presented as a limitation the lack of access to the full texts of some publications that were not available for online access, such as books and some articles. However, it is ­believed that there was no substantial shortcoming to the ­mapping ­carried out, thus keeping the justification of this study.

## CONCLUSION

Mapping the competencies of Forensic Nursing in disasters situations made it possible to reveal that the contributions of forensic nurses in this context is essential, since, due to the complexity of the event, the pyramid of basic human needs is ruptured, which culminates in biological damage, psycho-emotional and environmental factors that can lead to legal consequences. It was evident that it is necessary to invest in scientific studies through robust methods to consolidate the science of Forensic Nursing and promote greater clarity about the models of care of forensic nurses in the face of the phenomenon of disasters. It was concluded that approaching the issue proactively, with a view to preventing future risks for vulnerable people, families and communities and, interdisciplinary articulation with operators in the area of law have the potential to advance training and preparation strategies of these professionals in the face of the predictability of new events, since the risk of disasters is socially constructed, through a process related to the dynamics of development, which increasingly lacks professionals able to respond to such events.

It was identified that the forensic nurse acts in all phases of disasters, with the people, families and communities affected, using an interdisciplinary perspective. Thus, the development of competencies aimed at the practice of Forensic Nursing in disasters should be incorporated into the training strategies of these professionals in order to obtain better response standards. In short, the inclusion of forensic nurses within the scope of the disaster management continuum is an ethical-legal duty, including the specialty of Forensic Nursing as a social practice that provides the individual, family and community with compassionate and culturally sensitive care, sustained in the relationship of help to achieve security and social justice.
